# Triportal approach: endoscopic transorbital, transnasal and robotic transoral excision of parapharyngeal liposarcoma

**DOI:** 10.3171/2025.1.FOCVID24167

**Published:** 2025-04-01

**Authors:** Ben Chat Fong Ng, Wai-yin William Chung, Tsz-wai Lee, Wai-shun Ng, Tsun-cheong Chu, Hunter Kwok-lai Yuen, Calvin Hoi-kwan Mak

**Affiliations:** 1 Department of Neurosurgery, Queen Elizabeth Hospital, Hong Kong, China;; 2 Department of Ear, Nose, and Throat, Queen Elizabeth Hospital, Hong Kong, China; and; 3 Department of Ophthalmology and Visual Sciences, The Chinese University of Hong Kong, China

**Keywords:** endoscopic transorbital surgery, triportal, robotic transoral, liposarcoma, parapharyngeal space

## Abstract

A parapharyngeal space (PPS) tumor is a rare head and neck tumor. The choice of approach depends on the location, pathology, extent of invasion, and relationship with surrounding neurovascular structures. In this video, a PPS tumor was resected using a triportal approach: endoscopic transorbital, robotic transoral, and endoscopic transnasal surgery. Neurosurgeons, otorhinolaryngologists, and ophthalmologists jointly performed the operation. The pathology was a liposarcoma in the right upper parapharyngeal space, abutting the internal carotid artery. Postoperative endoscopy, MRI, and PET-CT showed no recurrence. The triportal approach is a novel technique in improving the exposure and multiangular dissection of PPS tumors with optimal protection to surrounding neurovascular structures.

The video can be found here: https://stream.cadmore.media/r10.3171/2025.1.FOCVID24167

## Transcript

I am going to present a case of triportal endoscopic transorbital, transnasal and robotic transoral excision of parapharyngeal liposarcoma.

### 0:30 Case History.

The patient is a 57-year-old man with good past health. He presented with recurrent otorrhea from the right for more than 10 years. Otoscope showed perforated tympanic membrane (TM). Pure tone audiogram showed 20–30 dB conductive hearing loss. Nasoendoscope showed a whitish firm mass obliterating the eustachian tube, and biopsy confirmed well-differentiated liposarcoma. After multidisciplinary meeting, we decided for radical excision due to poor sensitivity to radiosurgery and chemotherapy.

### 1:09 Imaging.

Preoperative MRI using as the neuronavigation is shown showing a 4-cm lipomatous lesion with internal enhancing septations at the right parapharyngeal space. The lesion abutted the right internal carotid artery posteriorly and displaced the lateral wall of nasopharyngeal mucosal medially.

### 1:31 Robotic Transoral Stage.

The first stage of the surgery began with the robotic transoral stage by the ENT surgeons, and this is the schematic diagram showing the setup of the da Vinci robot. In the robotic transoral stage, the patient was lying supine. An incision was made in the palate, and the palate was split to expose the tumor. Dissection of the anteroinferior aspect of the tumor was done transorally under neuronavigation.

### 2:06 Combined Transorbital and Transnasal Stage.

Then we moved to the second stage, the combined endoscopic transorbital and endonasal stage, and this is the schematic diagram showing the setup of this stage.

Lid crease incision was made on the right eye, and this is the schematic diagram showing the orientation of the endoscope. The greater wing of sphenoid was drilled to expose the middle fossa dura. Then, anterolateral triangle was identified between the V2 and V3, and drilling of the anterolateral triangle was started. And this is the schematic diagram showing the anterolateral triangle. After drilling of the anterolateral triangle, the tumor and the eustachian tube were exposed. And this is a schematic diagram showing the relationship of the tumor and the eustachian tube with V2 and V3. The cartilaginous portion of the eustachian tube was cut in order to mobilize and facilitate dissection of the superior aspect of the tumor while protecting the normal internal carotid artery. Then we moved to the combined endoscopic transorbital and endonasal dissection. In the endoscopic endonasal stage, the pterygoid plate and the posterior maxillary wall were drilled off. This is the schematic diagram showing the relationship of the tumor to the pterygoid plate and the pterygoid muscle. With the exposure and dissection of the transorbital and endonasal routes, the endonasal route finally communicated with the transorbital route to facilitate the dissection of the tumor. Back to the transorbital view, you can appreciate the cut end of the cartilaginous portion of the eustachian tube and the internal carotid artery. They were protected while the ENT surgeons dissected the tumor using the coblator in the endonasal route.

### 4:59 Final Stage: Robotic Transoral Stage.

At the final stage, the tumor was delivered in the robotic transoral route. And the palate incision was closed with a mucosal flap. The tumor was successfully excised en bloc.

### 5:30 Postoperative Care.

Postoperatively, the patient stayed in ICU for 2 days and had episodes of palatal bleeding and dehiscence requiring repair at postoperative 2 weeks’ time. He could start oral feeding with an obturator at 1.5 months. Pathology showed well-differentiated liposarcoma. There was no CSF leak, no visual impairment, and the right lid crease incision healed well.

### 5:57 Long-Term Follow-Up.

In long-term follow-up, the patient started radiotherapy at postoperative 1.5 months. He developed palatal dehiscence at postoperative 10 months requiring repair. There was no tumor recurrence at postoperative 1 year. The patient was on Ryle tube feeding postoperatively. An obturator was made at 1.5 months postoperatively, and the Ryle tube was removed. Later, the patient can tolerate an oral diet without an obturator with less nasal regurgitation. Postoperative MRI at 4 months and 1 year show no recurrence.

### 6:35 Discussion.

Liposarcoma of head and neck is a rare malignant soft tissue tumor derived from adipose tissue.^1^ Surgery in combination with radiotherapy is better than radiotherapy alone.^2–4^ Transitional approaches include transcervical, transparotid, and mandibulotomy. Disadvantages include malocclusion, loss of dentition, temporomandibular joint dysfunction and significant aesthetic impact, damage to the floor of the mouth leading to delay in oral feeding, need of tracheostomy, limited surgical exposure leading to possible tumor rupture, and neurovascular injury.^5,6^

### 7:16 Role of Endoscopic Transorbital Surgery.

In this case, endoscopic transorbital approach offered a minimally invasive approach to dissect the posterosuperior aspect of the tumor at the parapharyngeal space from the normal petrous internal carotid artery. It allows en bloc resection without mandibulotomy, and it is a highly versatile approach with portals. And this is the first case reporting a combination of three minimally invasive portals to excise head and neck region tumor.

### 7:48 Infratemporal Fossa: Transnasal Versus Transoribital.

The infratemporal fossa was bounded anteriorly by the posterior maxillary wall, posteriorly by the carotid sheath, superiorly by the greater wing of sphenoid, inferiorly by medial pterygoid muscle, medially by lateral pterygoid plate, and laterally by mandibular condyle and ramus. These are the slides to explain the differences between the transorbital and transnasal routes from the anatomical illustrations and cadaveric dissections. In the endoscopic transnasal route, the infratemporal fossa is opened in an anterior manner after drilling off the posterior wall of the maxillary sinus. This cadaveric dissection shows the relationship between the internal maxillary artery, V2, and descending palatine artery and nerve. While in the endoscopic transorbital route, the infratemporal fossa was opened superiorly by drilling of the greater wing of the sphenoid. This cadaveric dissection shows the relationship between the petrous ICA, eustachian tube, V2, and V3.

### 8:53 Conclusions.

In conclusion, complex skull base lesions often involve overlapping areas of different specialties. It requires a tailor-made approach to tackle the lesion. With the endoscopic transorbital approach, it significantly decreases the blood loss and decreases the length of stay. It also enables multiportal minimally invasive surgeries in order to achieve en bloc resection with margins while protecting the normal neurovascular structures.

**9:24 References^1–7^**

## Disclosures

The authors report no conflict of interest concerning the materials or methods used in this study or the findings specified in this publication.

## Author Contributions

Primary surgeon: W Ng, Mak. Assistant surgeon: BCF Ng, Lee, Yuen. Editing and drafting the video and abstract: BCF Ng, Chung, Lee. Critically revising the work: BCF Ng, Chung, Mak. Reviewed submitted version of the work: BCF Ng, Chung, Mak. Approved the final version of the work on behalf of all authors: BCF Ng. Supervision: BCF Ng, Chu, Mak.

## Supplemental Information

### Patient Informed Consent

The necessary patient informed consent was obtained in this study.
